# The effectiveness of a proposed counseling program in reducing mental wandering and digital stress among the students of the scientific departments in Afif, Shaqra University

**DOI:** 10.3389/fpsyg.2026.1739002

**Published:** 2026-04-22

**Authors:** Mohammed Hawwal Malfi Alotaibi

**Affiliations:** Shaqra University, Shaqraa, Saudi Arabia

**Keywords:** digital stress, effectiveness, learning outcomes, mental wandering, suggested counseling program

## Abstract

The research aimed at revealing the effectiveness of a proposed counseling program in reducing mental wandering and digital stress among students of the Scientific Departments in Afif, Shaqra University, Saudi Arabia. An intentional sample of students who showed high levels of mental wandering and digital stress in the exploratory study of the research problem was chosen. The number of the sample was (80) male and female students, who were divided into two groups, the first is the control group that was made up of 40 students and the other was the experimental group that consisted of 40 male and female students. The proposed counseling program was applied on the experimental group, and it consisted of (7) sessions. The research employed a descriptive approach and a quasi-experimental method. The study employed two instruments: the Digital Stress Scale, developed by the researcher, and the Mental Wandering Scale. The results of the study showed the effectiveness and positive impact of the proposed counseling program in reducing both mental wandering as well as digital stress among students of the experimental group compared to the control group and by post program measurement compared to the pre-measurement of the program. The results also revealed a direct correlation between digital stress and mental wandering. The research recommended the need to activate counseling and guidance programs to solve the technical and psychological problems to which the students are exposed, in addition to organizing courses and workshops for all employees in the educational sector to train them on the mechanisms of confronting the problems of students’ behaviors associated with this age of information and technology as an attempt to improve learning outcomes.

## 1 Introduction

At present, the world is witnessing rapid cultural and technological developments that have been reflected in the educational system. These developments require diversification and development of educational strategies in line with the requirements of digital transformation and prioritize the search for innovations in learning that attract and revolve around students, as well as exploring ways to address their technical and psychological problems with the aim of improving learning outcomes. Fink (2008) noted that modern developments reflected on the message and purpose of education, as it changed from improving the quality of education to improving the quality of learning, and the criteria for success changed from the quality of students entering the programs to the quality of graduate students, and the structures of learning and teaching also changed from covering the subject to specific learning outcomes, and this is consistent with Amer (2014) study, which confirmed that digital technology is changing the features of the educational system with its various elements, and had an impact on changing the role of the faculty member from a mere carrier of information to play the role of facilitator, clarifier, guide, trainer, evaluator and leader, and has also changed the role of the learner from a mere recipient of knowledge to the role of investigator, researcher and explorer.

Despite the benefits and advantages of using digital technology in education, it has a negative impact on users such as the digital stress and mind wandering phenomena.

Digital stress refers to the mental and emotional strain caused by the demands of using information and communication technologies (ICTs). Brod (1982, p. 754) was one of the pioneers who studied the phenomenon of digital stress and labeled it technostress. He defined the term as the mental and emotional challenges individuals face when attempting to adjust to the swiftly changing landscape of technological advancements. Wimmer and Waldenburger (2020, p. 1) describe digital stress as the emotional imbalance experienced when the demands of navigating digital technology outweigh an individual’s ability to adapt to them.

According to Reinecke et al. (2017), this type of stress often stems from the constant pressure to stay connected, manage multiple online tasks, and respond to continuous communication. Nick et al. (2022) added that digital stress can include feeling overwhelmed by a flood of notifications, the expectation to respond quickly, fear of judgment from others, and the overall pressure to stay engaged online. Zoghbi (2022) further explained that digital stress is a complex and dynamic experience involving cognitive, behavioral, and emotional responses. It often arises when individuals perceive digital interactions—such as emails, chat messages, app notifications, friend requests, and social media posts—as disruptive, exhausting, or threatening to their peace of mind and psychological well-being. Winstone et al. (2022) identified several common sources of digital stress, including passive internet browsing, wasting time online, exposure to harmful or upsetting content, receiving unwanted messages, feelings of guilt, pressure to appear perfect on social media, and fear of negative feedback or criticism in online spaces.

Digital stress arises from the reliance on social media platforms or virtual environments to fulfill unmet psychological needs. This often stems from frustrations related to real-life challenges, such as work and the pursuit of meaningful goals. It involves seeking validation, satisfaction, and connection with others to achieve a sense of psychological well-being. Consequently, individuals who experience this form of stress are at heightened risk of overusing information and communication technology (ICT) as a means of addressing these psychological needs. Numerous recent studies have established a link between the extensive use of digital technology and heightened feelings of anxiety and depression among users.

Moreover, such usage of digital technology has been shown to negatively affect various cognitive, psychological, and behavioral functions, which are critical components of the learning process (Abdul Raouf and Khlaiwi, 2022; Zoghbi, 2022). Numerous studies have demonstrated the detrimental effects of digital stress on individuals. For example, Ragu-Nathan et al. (2008) emphasized its negative impact on cognitive performance. Nick et al. (2022) explored how stress contributes to emotional exhaustion and social anxiety. Similarly, Reinecke et al. (2017) investigated the influence of digital stress on students, revealing its significant role in psychological burnout. These findings underscore the connection between stress and key psychological factors, all of which directly affect students’ academic performance and learning outcomes.

Studies by Zoghbi (2022) and Gharib et al. (2023) identified several key components of digital stress that can be summarized as follows:

Perceived Stress: This refers to the discomfort or distress people feel when they believe others expect them to always be available and responsive through digital platforms. It often includes feelings of guilt and anxiety.

1. Approval Anxiety: This is the psychological tension—mental, emotional, and behavioral—that comes from uncertainty about how others will react to one’s online content, such as posts, photos, messages, or even their personal profile.
2. Fear of Missing Out (FOMO) and Being Disconnected: This type of stress arises from the fear of missing socially rewarding experiences that others are having online while one is absent or not paying attention.
3. Communication Overload: This is the stress caused by being bombarded with too many digital inputs—such as notifications, messages, alerts, and comments—which can overwhelm a person and lead to mental exhaustion.

Several studies have explored how digital stress relates to various psychological and social factors. For instance, Gilbert et al. (2021) investigated the relationship between digital stress and aspects of internet vigilance—specifically, salience, interactivity, and monitoring—among university students. Their findings revealed that the prominence of digital interactions (how noticeable and unavoidable they are) was most strongly linked to perceived digital stress. The other two dimensions, interactivity and monitoring, had weaker, indirect connections to digital stress, mainly through the burden of excessive communication. Abdul Raouf and Khlaiwi (2022) examined digital stress among 1,057 Saudi university students. Their study found differences based on academic majors, with students in humanities experiencing more digital stress than those in scientific disciplines. Zoghbi (2022) aimed to develop a digital stress scale and analyze its underlying structure. The study identified six key components of digital stress: high communication load, multitasking, availability stress, social approval anxiety, hyper-alertness and fear of missing out, and concerns over privacy and trust.

Misra and Stokols (2012) confirmed the impact of internet use on digital stress in students.

In another study, Gharib et al. (2023) explored the structural relationship between digital stress, academic resilience, i.e., students’ ability to cope with academic challenges, and university integration. They found that higher levels of digital stress were negatively associated with both academic resilience and students’ sense of belonging at university. Additional studies have shown that digital stress is significantly linked to a range of emotional and social outcomes. Wrede et al. (2021) found correlations between digital stress and feelings of acceptance, popularity, social anxiety, rejection sensitivity, loneliness, and depression. Nick et al. (2022) highlighted the growing concern over the risks of digital stress, while Hall et al. (2021) pointed to its connection with psychological and social distress. Groote and Ouytsel (2022) also found that digital stress plays a role in friendship dynamics among youth.

Students’ academic performance is closely tied to the brain’s cognitive processes, which process a range of stimuli—both academic and non-academic. This influx of ideas can overwhelm the mind, leading to partial or complete distraction from the primary task at hand. Psychologists refer to this phenomenon as mental wandering, which can obstruct the acquisition of knowledge and skills. Consequently, mental wandering poses a significant barrier to students in achieving their academic goals (Biederman et al., 2019, p. 16).

Mind wandering refers to the fluctuation of attention, influenced by various situations, the significance of those situations to the individual, and the degree of cognitive engagement with them. According to Seli et al. (2018, p. 1250) the extent of wandering depends on the complexity or simplicity of the tasks occupying the mind. Similarly, Al-Feil (2019) defines mind wandering as an involuntary shift in focus from the primary task to internal or external thoughts, which may or may not be related to the initial activity.

Mental wandering is a significant factor influencing students’ academic performance during the learning process. It has recently gained considerable attention from psychologists and educators due to its impact and the potential risks it poses to students’ achievement and educational outcomes. According to Randall et al. (2014, p. 1425) mental wandering disrupts learners’ ability to complete required tasks, particularly academic ones, often leading to personal challenges. These challenges result in deficits in both academic and non-academic liabilities, leaving learners in a state of cognitive distraction and exposing them to negative consequences. Similarly, Al-Masry (2022, p. 306) emphasizes these harmful effects.

Vago and Zeidan (2016, p. 100) noted that mental wandering causes include some psychological disorders such as attention deficit disorders, working memory disorders, and distractions in the external environment surrounding the student. Several previous studies have highlighted how common mental wandering is among undergraduate students. For instance, Hollis (2013) found that levels of mental wandering could predict both academic performance and how interested students were in the subject matter. Rahl et al. (2017) showed that mindfulness training can be effective in helping students reduce mental wandering. Similarly, Al-Feil (2018) explored the use of a scenario-based learning model and found it helped decrease mental distractions during learning. Al-Omari et al. (2019) conducted a study on a program that used diffuse learning techniques. They found this approach not only improved learning outcomes but also reduced mental wandering, with the positive effects continuing over time. Pan et al. (2020) focused specifically on online learning, showing that mental wandering can significantly reduce attention during virtual lectures. Finally, Al-Sawaf (2022) demonstrated that using e-active learning strategies and electronic discussions through online platforms was effective in keeping students more mentally engaged.

In the Saudi context, a number of studies showed the negative impact of mental wandering and digital stress on the academic performance of students. Al-Masry (2022, p. 299) indicated that there is a relationship between mental wandering and academic underachievement. Gharib et al. (2023) confirmed that there is a negative relationship between mind wandering and academic performance.

The studies of Shepherd (2019), Al-feil (2018), Al-Omari et al. (2019), and Al-Maraghi (2020) identify several key causes of mental wandering that can be summarized as:

1. Limited mental capacity arises from the interplay between memory functions and the demands of a task.
2. Tasks requiring continuous attention and force the mind to seek for escape from the pressures associated with prolonged concentration.
3. Negative moods contribute to a higher tendency for mental wandering compared to positive emotional states, particularly when engaging in tasks.
4. Negative thoughts about the future often arise when students worry about upcoming challenges.
5. Negative expectations that might include stress, lack of sleep, overwhelming responsibilities, or mental overload which can cause their thoughts to drift away from the task at hand.
6. Positive expectations such as feelings of joy, excitement, improved focus, or even simply noticing things in the environment that can also become distractions, pulling people’s attention away from what they are supposed to be doing.
7. Deep and complex expectations related to tasks that demand planning, critical thinking, and decision-making. These activities push students to challenge themselves and test their capabilities, which can be both engaging and mentally demanding.

Due to the importance of the undergraduate stage in the lives of students, that prepares them for their future roles in society, the current research focuses on their problems resulting from modern technology. It designs a counseling program to reduce the levels of digital stress among Shaqra University students, which can be one of the reasons for mental wandering within lectures, which affects the academic achievement of students and the outcomes of the educational process. It evaluates the effectiveness of the program to reduce mental wandering. Moreover, it explores the relationship between mental wandering and digital stress, a variable not commonly addressed in previous research.

This study aims at answering the following questions:

1. How effective is the proposed counseling program in reducing the level of mental wandering among subjects of the study?
2. How effective is the proposed counseling program in reducing the level of digital stress among students?
3. Is there a relationship between mental wandering and digital stress among subjects of study?

To answer the research questions, the research hypotheses were formulated as follows:

1. There are differences between the average scores of the study’s control group and the experimental group in the dimensional measurement of the mental wandering scale, in favor of the experimental group.
2. There are differences between the average scores of the experimental group in the pre- and post-measurement of the mental wandering scale, in favor of the post-measurement.
3. There are differences between the average scores of the control group and the experimental group in the dimensional measurement of the digital stress scale, in favor of the experimental group.
4. There are differences between the average scores of the experimental group students in the pre- and post-measurement of the digital stress scale, in favor of the post-measurement.

The current research contributes to assisting university students to deal with their psychological and technical problems, which contributes to improving their academic achievement and learning outcomes. It is an addition to studies about mental wandering and digital stress a topic that is not addressed widely in the Arab World context. It is expected to benefit educational institutions by providing training and counseling programs, to solve the educational, technical and psychological problems related to mental wandering and digital stress facing students in different educational stages. It may contribute to improving learning outcomes by guidance for the use of appropriate strategies and learning methods to eliminate students’ educational, psychological, and technical problems related to mental wandering and digital stress. Moreover, it proposes measurement tools (for digital stress, mental wandering), which can be used to diagnose students’ problems. The results of the research can be used in designing counseling programs that develop academic achievement and integration and develop positive attitudes for students toward the optimal use of digital technologies.

The study starts by providing an introduction that reviewed relevant literature about the topic. Then, it presents the methods used including data collection, description of the sample, research procedures, program validity and reliability, description of the program and the amendments made to it. Followed by, results, interpretation and discussion. Finally, a conclusion section is added as well as references.

## 2 Methods

The current research utilizes the descriptive approach. It employs the description and interpretation of what an object is, the diagnosis of the prevailing practices used in the current research in the preparation of theoretical frameworks for mental wandering and digital stress, and the description of the proposed mentoring program. It also uses quasi-experimental approach, i.e., the use of experiment in measuring and adjusting different variables to identify the impact of the independent variable on the dependent variable, and the current research variables are the independent variable, which is the proposed counseling program, and the dependent variable, which is mental wandering, digital stress.

### 2.1 Experimental design of the study

**Figure 1:**
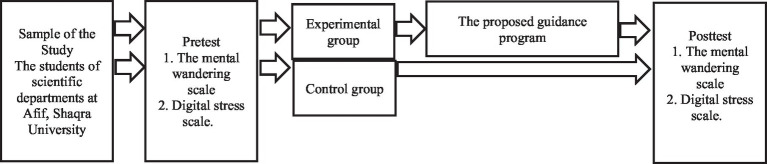
The experimental design of the research.

The study followed the experimental design of Pretest-Posttest Control Group Design. i.e. the control and experimental group system, and the pre- and post-application of research tools (McMillan and Schumacher, 1993, p. 328) as illustrated in Figure 1.

### 2.2 Research procedures

This study involved several steps in both its design and fieldwork, which are outlined.

#### 2.2.1 Research population and sample

##### 2.2.1.1 The exploratory sample

To better understand the research variables-digital stress and mental wandering-an exploration sample was drawn from students in the scientific departments at Afif campus. This group included 128 male and female students and was used to help diagnose and clarify the main variables of the study.

##### 2.2.1.2 The basic group

Based on the results of the exploration study, the main sample was intentionally chosen from students who scored high on both the mental wandering and digital stress scales. This core group included 80 male and female students, making up 62.5% of those who showed high levels in the initial screening.

These 80 students were then divided into two groups:

- Control group: 40 students
- Experimental group: 40 students

Table 1 shows the frequency and relative distribution of the basic research sample.

**Table 1:** Frequency and relative distribution of demographic and academic characteristics of the study sample according to the selection criteria.

Variable	Description	Number	Percentage	Variable	Description	Number	Percentage
Gender	Female	93	72.6%	CGBA	Excellent	18	14.1%
	Male	35	27.3%		Very good	53	41.4%
Department	Science	33	24.8%		Good	41	32%
	Computer	5	3.9%		Pass	15	11.7%
	Law	85	66.4%		Failed	1	0.8%
	English	5	3.9%				

##### 2.2.1.3 Preparation of research tools: the researcher used the following research tools using the current research variables

##### 2.2.1.4 Digital stress scale

The researcher designed the digital stress Scale following these steps:

1 *Determining the goal of the scale*: The aim of the scale is to diagnose and detect digital stress, its dimensions and behaviors, which appear on the behaviors of subjects of the study.
2 *Preparation of the scale*: Some of the previous studies that dealt with measuring the level of digital stress were reviewed, such as the study of Gilbert et al. (2021), Zoghbi (2022), Gharib et al. (2023), and Abdul Raouf and Khlaiwi (2022), then the scale was prepared in its initial form which consist of 5 main axes, namely (digital social acceptance anxiety, cyber risks, availability stress, multitasking, fear of losing information and communication), The number of items of the scale are 27 items, and their distribution are shown in the Table 2.

**Table 2:** Distribution of the digital stress scale items on the axes.

No.	Description of axis covered by the scale	Number of items	Percentage
1	Digital social acceptance anxiety	9	33.3%
2	Cyber risks	6	22.2%
3	Availability stress	5	18.5%
4	Multitasking	3	11.2%
5	Fear of losing information and communication	4	14.81%
Total		27	100%

It is clear from Table 2 that the axis of digital social acceptance anxiety represents the highest percentage of the scale’s items (33.3%), followed by the cyber risk axis (22.2%), which reflects the scale’s focus on the social dimensions of digital stress.

3 *Determining the degree of response on the scale*: The scale is designed using a five points Likert scale (i.e., Strongly Apply = 5 points, Apply = 4 points, Apply to some extent = 3 points, Not applicable = 2 points, Not applicable at all = 1 point). Each student is asked to choose only one response, so the total score of the Digital Stress Scale is the highest score = 135 and the lowest score is 27. As shown in Table 2.

##### 2.2.1.5 The psychometric properties of the digital stress scale

To make sure the scale is reliable and valid, it was first tested with a separate group of 20 students who were not part of the main research sample. This helped the researcher check both the accuracy and consistency of the scale.

##### 2.2.1.6 Validity of the digital stress scale

The researcher verified the validity of the scale in several ways.

1 Face validity

The initial version of the digital stress scale was shared with a panel of experts in educational and psychological sciences. They reviewed the scale, giving feedback on whether the items were clear, well-worded, and suitable for each section, as well as whether the instructions were easy to understand. Based on their suggestions, the researcher made some adjustments to improve the scale. In the end, 85% of the experts agreed that the scale was appropriate and ready for use.

2 Structural validity

To check how well the scale holds together, the researcher looked at its internal consistency. This involved calculating the correlation between each item and its corresponding dimension, as well as between each item and the overall scale. Table 3 shows Pearson correlation coefficients between the items of each dimension and the overall score of the scale (internal consistency truthfulness).

**Table 3:** The values of the internal consistency coefficients of the items and axes of the digital stress meter.

Dimension	Item no.	Item correlation with axis as a whole	Item correlation with scale	Significance
1. The anxiety of digital acceptance	1	0.666**	0.474*	Significant
	2	0.885**	0.907**	Significant
	3	0.805**	0.745**	Significant
	4	0.857**	0.685**	Significant
	5	0.890**	0.766**	Significant
	6	0.792**	0.769**	Significant
	7	0.884**	0.904**	Significant
	8	0.865**	0.856**	Significant
	9	0.866**	0.941**	Significant
2. Cyber risks	10	0.910**	0.789**	Significant
	11	0.909**	0.916**	Significant
	12	0.919**	0.829**	Significant
	13	0.949**	0.812**	Significant
	14	0.928**	0.873**	Significant
	15	0.933**	0.916**	Significant
3. Availability stress	16	0.882**	0.843**	Significant
	17	0.956**	0.882**	Significant
	18	0.938**	0.873**	Significant
	19	0.926**	0.852**	Significant
	20	0.876**	0.937**	Significant
4. Multitasking	21	0.919**	0.903**	Significant
	22	0.953**	0.864**	Significant
	23	0.981**	0.919**	Significant
5. Fear of losing information	24	0.918**	0.895**	Significant
	25	0.928**	0.858**	Significant
	26	0.934**	0.866**	Significant
	27	0.792**	0.784**	Significant

** Significant at the level of (0.01) * Significant at the level of (0.05).

The results of Table 3, showed that the values of the correlation coefficients between the items of the scale and the axis, as well as between the items and the scale as a whole, are mostly significant (**) at the level of significance (0.01), which shows the validity of the construction of the scale.

Pearson’s correlation coefficient was also calculated for the items of the scale and the scale as a whole, and it was shown that the correlation coefficient for the digital social acceptance anxiety dimension = 0.948, the cyber risk dimension = 0.927, availability stress = 0.957, multitasking = 0.939, fear of losing contacts = 0.953. It was also found that all values are significant (**) at the level of (0.01), which ensures the validity of the internal consistency of the scale.

3 Discriminant validity

The discriminant validity of the Digital Stress Scale was tested using an exploratory sample of 20 students, separate from the main study sample. This was done by examining the significance of the differences between the mean scores of the highest 27% and the lowest 27% of the participants, according to the discriminant validity formula, for each dimension of the scale as well as the total scale score. The (t) value was calculated to determine the significance of these differences, as shown in Table 4.

**Table 4:** The significance of the differences between the upper and lower groups on the dimensions of the digital stress scale.

Dimension	Highest group average	Standard deviation	Lower group average	Standard deviation	T value	Significance
1. The anxiety of digital acceptance	42.33	3.88	22.17	7.65	5.757	0.01
2. Cyber risks	28.50	2.51	13.50	6.38	5.359	0.01
3. Availability stress	24.50	0.84	10.50	3.89	8.627	0.01
4. Multitasking	14.67	0.52	6.00	2.10	9.827	0.01
5. Fear of losing information	18.33	1.86	7.83	2.93	7.414	0.01
Overall score of the scale	127.83	6.43	62.00	21.08	7.317	0.01

(*) The tabulated *t*-value at 10 degrees of freedom and a significance level of 0.01 is 3.169.

Table 4 shows that there are statistically significant differences at the level of (0.01) between the average scores of the upper and lower groups in all dimensions of the digital stress scale and the total score of the scale, in favor of the upper group, which indicates the ability of the scale to distinguish between the high and low scores of the digital stress, and confirms its discriminatory validity.

4 Scale reliability

The researcher calculated the reliability of the Digital Stress Scale using two methods:

(i) Cronbach’s alpha coefficient

The stability of the scale was calculated using the Alpha equation and the alpha coefficient of the axes of the digital stress scale as follows: The first dimension: digital social acceptance anxiety was (0.944), the second dimension: cyber risks amounted to (0.965), the third dimension: availability stress (0.952), the fourth dimension: multitasking calculated at (0.944), and the fifth dimension: fear from loss of information and communication amounted to (0.916), while the Cronbach alpha coefficient for the digital stress scale scored a total value of (0.984), which is ‘too high’ and indicates that the digital stress meter has high stability, which means that the scale is valid.

(ii) The split-half method

Reliability was also calculated using the split-half method for the items of the Digital Stress Scale. The correlation coefficient between the odd and even items was found to be 0.990. After correcting the coefficient using the Spearman-Brown formula, it was 0.995, which is also a very high value. This demonstrates that the scale has a high degree of reliability, making the Digital Stress Scale a valid research tool.

##### 2.2.1.7 Preparation of the final draft of the scale

After confirming the validity of the scale, it has been designed electronically via Google Forms and provided to students of the study sample in the Supplementary file 1.

##### 2.2.1.8 Preparation of the mental wandering scale

The current study is based on the mental wandering scale prepared by Al-Feil (2018), which was made up of two axes, namely (mental wandering related to the topic of the lecture, and mental wandering not related to the topic of the lecture) and consisted of 26 items. They were distributed on the scale’s axes as shown in Table 5.

**Table 5:** The distribution of the items of the mental wandering scale.

No.	Axes of the mental wandering scale	No. of items	Relative weight
1	Mental wandering related to the topic of the lecture	12	46.2%
2	Mental wandering unrelated to the topic of the lecture	14	53.8%
Total		26	100%

### 2.3 Measurement of responses

Students responded to the Mind Wandering Scale using a three-point Likert scale: “Always” (3 points), “Sometimes” (2 points), and “Never” (1 point). Each student was asked to choose just one response per item. This means the total possible score on the Mind Wandering Scale ranges from a minimum of 26 to a maximum of 78, as shown in Table 6.

**Table 6:** Correlation coefficients of the paragraphs of the mental transformation scale with the dimension to which they belong and the total score of the scale (the validity of internal consistency).

Axes of the mental wandering scale	Item no.	Item correlation with axis as a whole	Item correlation with scale	Significance
1. Mental wandering related to the topic of the lecture	1	0.809^**^	0.619^**^	Significant
	2	0.815^**^	0.645^**^	Significant
	3	0.815^**^	0.638^**^	Significant
	4	0.607^**^	0.552^*^	Significant
	5	0.824^**^	0.644^**^	Significant
	6	0.814^**^	0.586^**^	Significant
	7	0.740^**^	0.524^*^	Significant
	8	0.766^**^	0.542^*^	Significant
	9	0.593^**^	0.652^**^	Significant
	10	0.823^**^	0.619^**^	Significant
	11	0.724^**^	0.625^**^	Significant
	12	0.869^**^	0.615^**^	Significant
2. Mental wandering unrelated to the topic of the lecture	13	0.742^**^	0.709^**^	Significant
	14	0.865^**^	0.819^**^	Significant
	15	0.866^**^	0.739^**^	Significant
	16	0.629^**^	0.645^**^	Significant
	17	0.878^**^	0.791^**^	Significant
	18	0.870^**^	0.859^**^	Significant
	19	0.919^**^	0.809^**^	Significant
	20	0.823^**^	0.716^**^	Significant
	21	0.909^**^	0.788^**^	Significant
	22	0.872^**^	0.838^**^	Significant
	23	0.857^**^	0.733^**^	Significant
	24	0.726^**^	0.655^**^	Significant
	25	0.955^**^	0.859^**^	Significant
	26	0.811^**^	0.713^**^	Significant

#### 2.3.1 Psychometric properties of the mind wandering scale

The researcher calculated the psychometric properties of the Mind Wandering Scale to further standardize it and ensure it was suitable for use with the current research sample. The scale was administered to a pilot group of 20 students, separate from the main research sample, to assess its validity and reliability.

#### 2.3.2 Assessing the validity of the mind wandering scale

The validity of the scale was checked in several ways, including:

1 Face validity

The initial version of the scale was reviewed by a panel of experts specialized in in educational and psychological sciences. They provided feedback on its appropriateness, clarity, and wording. Based on their suggestions, the scale was revised. Then the experts agreed that the scale was suitable for use, with a face validity rating of 90%.

2 Construct validity

Internal consistency was assessed by calculating the correlation between each item and its corresponding dimension, as well as the correlation between each item and the total scale score. Table 6 shows the correlation values for the items on the Mind Wandering Scale.

The results of Table 6 show that the correlation coefficients of all paragraphs with the dimension to which they belong and the total score of the scale were positive and statistically significant at the level of (0.01), which indicates that the mental transformation scale has a high degree of internal consistency.

Pearson’s correlation coefficient was also calculated to assess the internal consistency of the scale’s dimensions and the scale as a whole. The correlation for the “mind wandering related to the lecture topic” axis was 0.792, and for the “mind wandering unrelated to the lecture topic” axis, it was 0.910. Both correlations are significant (**) at the 0.01 level, confirming the internal consistency validity of the scale.

3 Discriminant validity

The discriminant validity of the Mind Wandering Scale was tested using the pilot sample of 20 students who were not part of the main research sample. This was done by examining the differences between the mean scores of the highest 27% and the lowest 27% of participants, according to the discriminant validity formula, for each dimension and for the overall scale. The t-value was calculated to determine the significance of these differences, as shown in Table 7.

**Table 7:** The significance of statistical differences between the lowest and highest degrees (27%) to identify discriminatory ability for the mental wandering scale.

Scale axes	Highest group average	Standard deviation	Lower group average	Standard deviation	*t* value	Significance
Mental wandering related to the topic of the lecture	A35.3333	0.81650	21.8333	4.83391	6.745	0.01
Mental wandering not related to the topic of the lecture	40.5000	2.50998	19.8333	5.60060	8.248	0.01
Total	73.3333	5.88784	44.8333	9.66264	6.170	0.01

The tabular value of (t) at degrees of freedom (10) and significance level (0.01) is 3,169.

The results of Table 7 indicate that there are statistically significant differences at the level of (0.01) between the average scores of the upper and lower groups in all dimensions of the Mental Transformation Scale as well as in the total score of the scale, which confirms the validity of the discriminatory scale and its validity to apply to the study sample.

#### 2.3.3 Calculating scale reliability

The researcher assessed the reliability of the Mind Wandering Scale using two methods:

(i) Cronbach’s alpha coefficient

The reliability of the scale was assessed using Cronbach’s alpha. The results showed a high level of consistency. The alpha coefficient for the first dimension-mind wandering related to the lecture topic was 0.934, while the second dimension-mind wandering unrelated to the lecture topic-scored even higher at 0.967. Overall, the total alpha coefficient for the mind wandering scale was 0.956. These high values indicate that the scale is highly reliable, giving us confidence in both its accuracy and its use.

(ii) The split-half method

Reliability was also assessed using the split-half method, by dividing the scale items into odd and even groups. The correlation coefficient was found to be 0.947, and after adjustment using the Spearman-Brown formula, the reliability coefficient reached 0.973. This is also of high value, confirming that the scale has a high degree of reliability. Therefore, the Mind Wandering Scale can be confidently used with the main research sample.

#### 2.3.4 Preparation of the final version of the scale

After confirming the scale’s suitability for use, the final version was created electronically using Google Forms and made available to the main research sample in the Supplementary file 1.

#### 2.3.5 Designing the experimental intervention: (the proposed counseling program)

The researcher developed and structured the proposed program according to the following stages:

1 Stage one

Analysis is the first step in building the counseling program and it includes the following steps:

(i) Defining the program’s philosophy

The proposed program is based on counseling activities aimed at reducing mind wandering and digital stress.

(ii) Identifying the rationale for developing the counseling program

The rationale for proposing the program is as follows:

(a) Identifying the behavioral, cognitive, and technological problems exhibited by the college students, specifically mind wandering and digital stress, which are addressed in the current research.
(b) Providing guidance, instructions, and strategies to help reduce mind wandering behaviors and digital stress.

(iii) Analyzing the characteristics and needs of the target group

The target group for this study consists of undergraduate male and female students from the scientific departments at the College of Science and Humanities in Afif. These students are young adults, aged between 19 and 25, and come from a variety of scientific and humanities majors. They were selected because they demonstrated high levels of mind wandering during lectures as well as elevated levels of digital stress.

(iv) Defining the foundations of the counseling program

The proposed program in this study is based on the following foundations:

1 Cognitive foundations

These are reflected in providing scientific content based on guidance and instructions to help students reduce behaviors related to mind wandering and digital stress.

2 Technological foundations

This involves the proper use of technological tools to help decrease digital stress.

3 Psychological foundations
4 Social foundations

The program considers students’ psychological needs and individual differences, presenting information in various ways and using enrichment activities within the program.

These are demonstrated by encouraging social interaction among students through discussion groups focused on strategies to reduce mind wandering and digital stress, as well as involving them in group activities within the program.

2 Stage two: design

This stage includes:

(i) Defining the educational objectives of the proposed program

The general objectives of the program are as follows:

1. To identify student behaviors that impact learning outcomes.
2. To enrich students’ knowledge and experiences with effective solutions to address the behavioral challenges they face.
3. To reduce instances of mind wandering among students during lectures.
4. To decrease digital stress among students, which can negatively affect learning outcomes.

Based on these goals, 20 specific behavioral objectives were developed for the program. These objectives describe what is expected from the research sample after participating in the counseling program, and they are distributed across the main topics of the program’s scientific content, as shown in Table 8.

(ii) Defining the educational content

**Table 8:** Behavioral objectives of the proposed program content and their relative weight for each topic.

No.	Topics	Number of behavioral goals	Behavioral goals	Percentage
1	Memory, mental attention and distractions	3	Learn about memory types and functions. Use strategies to enhance focus and attention. Identify sources of distraction and ways to reduce them	15%
2	Strategies to reduce mental wandering	4	Understand the concept of mental wandering and its causes. Identify the mechanisms of coping with mental wandering. Practice mindfulness techniques. Employ prevention strategies to prevent mental wandering during learning.	20%
3	Mechanisms of positive use of technology	3	Distinguish between positive and negative use of technology. Learn about the evolution of technology and its impact on learning. Positively apply technology to enhance digital learning and interaction.	15%
4	Causes and symptoms of digital stress	5	Identify the causes of digital stress. Identify the components of digital stress. Measuring the impact of digital stress on mental performance. On the physical and psychological symptoms of digital stress. Analyze the relationship between technology use and digital stress	25%
5	Digital stress reduction strategies	5	Organize digital tasks effectively. Maintaining reliability in digital transactions. Managing digital interactions to reduce stress. Organize the digital availability and availability of tasks. Enhance cyber protection while using digital devices.	25%
Total		20		100%

The scientific content of the counseling program was carefully selected with the expectation that it would help reduce mind wandering and digital stress among the research sample. The researcher ensured that the content was comprehensive, integrated, and logically sequenced and connected across topics. The program topics were distributed over five counseling sessions, with scientific content provided for each session. In addition, there were two extra sessions: one for introductions and another for concluding the program and administering research tools. Table 9 shows how the scientific content was distributed across the program sessions, along with the number of objectives and activities included with each topic.

(iii) Identifying the program’s interactive activities

**Table 9:** Distribution of program content and enrichment activities to the sessions of the proposed mentoring program.

Session	Title	Scientific content	Educational strategies	Educational technologies	Duration
First session	Introduction	Introduction of the researcher and the research sample	Brainstorming	E-Questionnaire	90 min
Second session	mental attention and its distractions	Administering the research tools as a pre-test	Dialog and discussion	Interactive presentations	90 min
Third session	Strategies to reduce mental wandering		E-Learning	Mind maps blogs	90 min
Fourth session	Mechanisms of positive use of technology	Memory and its types.	Brainstorming	Interactive presentations	90 min
Fifth session	Causes and symptoms of digital stress	Mechanisms of memory enhancement	Speech and dialog	Mind maps blogs	90 min
Sixth session	Digital stress reduction strategies	Distractions	Learning stations	Interactive presentations	90 min
Seventh session	Conclusion and Assessment	Attention-grabbing mechanisms	Self-learning	Mind maps blogs	90 min

The interactive enrichment activities for the proposed counseling program were designed by specifying the objective of each activity according to the scientific content of the guidance session, identifying the requirements for implementing each activity and the time needed, and outlining the specific instructions for each activity. The activities were structured to ensure both individual and group interaction among the research sample.

3 Third stage: evaluation

The evaluation methods used in the current counseling program are as follows:

(i) Pre-evaluation

This involved diagnosing mind wandering and digital stress among the basic research sample.

(ii) Formative evaluation

This is continuous evaluation conducted throughout the implementation of the program’s guidance sessions. It includes feedback, enrichment tasks, interaction during interactive presentations, and the execution of training and enrichment activities.

(iii) Final evaluation

This involved reapplying the research tools after the completion of the counseling program.

4 Fourth stage: scientific validation of the proposed guidance program

After completing the preparation of the proposed program, content validity was assessed by presenting the program to a group of experts in the fields of education and educational psychology. This was done to ensure the soundness of its structure and to verify the appropriateness of the following aspects:

1. Clarity of the program’s general and behavioral objectives.
2. Suitability of the program for the target.
3. Logical sequencing of the scientific content of the program’s sessions.
4. Appropriateness of the enrichment activities and their implementation methods.
5. Suitability of the teaching strategies and techniques for the counseling program.
6. Adequacy of the program’s timeline for implementation.
7. Appropriateness of the program’s evaluation tools.

The experts provided some suggestions for modifications which the researcher implemented. Using an agreement rate calculation formula, it was found that the level of agreement among the experts on the validity of the program was 90%. This high level of agreement indicates that the proposed counseling program is suitable for practical application and for measuring its outcomes.

5 Field experimentation

After the design and production of the counseling program were completed and its suitability for application was confirmed, the field experiment was conducted on the main research sample in three stages:

(i) Pre-experiment stage

An introductory session was organized for the sample totaling 80 students. The students were divided equally into two groups: the first is experimental and the other is a control group, with 40 students in each. A pre-test was conducted for both groups using the mind wandering and digital stress scales to ensure the equivalence and homogeneity of the groups. An independent samples *t*-test was used for this purpose, as shown in Table 10.

**Table 10:** Test results of the differences between the average scores of the control and experimental groups in the pre-measurement of the two research groups.

Scale	Group	No.	Mean	Standard deviation	df	*t*	*p*	Significance
Mind wandering	Control	40	57.5000	12.72591	78	0.598	0.551	Not statistically significant
	Experimental	40	55.8500	11.93476	78	0.598	0.551	Not statistically significant
Digital stress	Control	40	91.9500	29.81563	78	1.484	0.142	Not statistically significant
	Experimental	40	82.9750	23.95882	78	1.484	0.142	Not statistically significant

Table 10 above demonstrates that the calculated *t*-value is less than the critical *t*-value, as the critical *t*-value at a degree of freedom of 78 and a significance level of 0.01 is 3.106. This indicates that there are no statistically significant differences between the experimental and control groups on the mind wandering and digital stress scales in the pre-test. Therefore, it can be concluded that the two research groups were equivalent prior to the experiment.

(ii) Experiment implementation stage

After conducting the pre-test and confirming the equivalence of the two groups, the researcher proceeded with the application of the program to the research groups as follows:

(a) Experimental group

The students in the experimental group were introduced to the proposed guidance program, its objectives, and its sessions.

(b) Control group

The control group did not receive any orientation about the program.

(iii) Experiment evaluation stage

After completing the implementation of the experiment and evaluating the program and its impact, a post-test was conducted using the two scales for both the control and experimental groups. The responses were recorded, analyzed, and the results interpreted to develop research recommendations and propose future studies.

## 3 Research results, interpretation, and discussion

After collecting and recording the responses of the research sample, the data were statistically analyzed using the Statistical Package for the Social Sciences (SPSS), version 26. Below is a presentation of the research results, addressing the study’s questions and testing its hypotheses as follows:

### 3.1 The first hypothesis

The first hypothesis states: “There are differences between the mean scores of students in the experimental and control groups in the post-test measurement for reducing mind wandering when using the proposed guidance program”.

To test this hypothesis, the research data were described and summarized by calculating the mean and standard deviation, as well as the T-value for independent samples (Independent-Samples *t*-Test). The results are presented in Table 11.

**Table 11:** Descriptive statistics and *t*-value showing the significance of the differences in the post-test mean scores between the experimental and control groups on the mind wandering scale, along with the calculation of Eta squared.

Dimensions of the mental wandering scale	Group	Number	Arithmetic mean	Standard deviation	Degree of freedom	*t*- test value	Statistical significance	Eta square	Effectiveness level
Mental wandering related to the topic of the lecture	Control	40	28.35	5.83	78	3.221	Statistically significant (0.01)	0.140	Great impact
	Experimental	40	23.20	8.65					
Mental wandering not related to the topic of the lecture	Control	40	28.52	8.74	78	4.247	Statistically significant (0.01)	0.188	Great impact
	Experimental	40	20.65	7.81					
The scale as a whole	Control	40	62.27	13.61	78	5.539	Statistically significant (0.01)	0.282	Great impact
	Experimental	40	46.87	11.12					

Table 11 shows that the mean score of the experimental group on the mind wandering scale was 46.88 with a standard deviation of 11.12 out of a total possible score of 78. This is lower than the mean score of the control group, which was 62.27 with a standard deviation of 13.61-a difference of 15.40 points. This indicates that there are differences between the mean post-test scores of the control and experimental groups on the mind wandering scale, in favor of the experimental group. In other words, the program led to a reduction in mind wandering among the experimental group compared to the control group.

To verify the significance of these differences between the mean, the T-value for the two independent groups of equal size was calculated, as shown in Table 11. The calculated T-value was found to be higher than the critical T-value at the 0.01 significance level and 78 degrees of freedom, where the critical T-value is 3.106. This demonstrates that there are statistically significant differences in all dimensions of the mind wandering scale and in the total scale score. This supports the acceptance of the hypothesis stating that “there are statistically significant differences between the mean post-test scores of the control and experimental groups on the mind wandering scale after implementing the guidance program, in favor of the experimental group.” This indicates a reduction in mind wandering among the experimental group students.

To determine the effect size, Eta squared (*η*^2^) was calculated to assess the impact of the independent variable on the dependent variable. The value for the overall scale was 0.28, which means that 28% of the variance between the mean mind wandering scores of the control and experimental groups is attributable to the experimental intervention. The Eta squared values exceeded 0.14 for all dimensions of the scale, as shown in Table 11, indicating a large effect of the independent variable on the dependent variable, which is the reduction of mind wandering.

This suggests that there was a significant and noticeable decrease in the post-test scores compared to the pre-test scores across all dimensions of the mind wandering scale and for the scale as a whole. This reduction can be attributed to the experimental intervention, namely the implementation of the proposed guidance program. Thus, the program had a substantial and effective educational impact, providing strong scientific evidence for its use in reducing mind wandering-both to related and unrelated to the lecture topic wandering-among a sample of students from the scientific departments at the College of Science and Humanities in Afif.

### 3.2 The second hypothesis

The second hypothesis states: “There are differences between the mean pre-test and post-test scores of the experimental group on the mind wandering scale when using the proposed guidance program”.

To test this hypothesis, the research data were described and summarized by calculating the mean and standard deviation, as well as the T-value for paired samples (Paired-Samples *t*-Test). The results are presented in Table 12.

**Table 12:** Descriptive statistics and *t*-test results show the significance of the differences between the pre-test and post-test scores of the experimental group on the mind wandering scale, along with the calculation of effect size (d).

Dimensions of the mental wandering scale	Group	Number	Arithmetic mean	Standard deviation	Degree of freedom	*t*- test value	Statistical significance	Effect size (d)	Effectiveness level
Mental wandering related to the topic of the lecture	Pre-test	40	27.57	6.10	39	3.619	Statistically significant (0.01)	0.60	Medium impact
	Post-test	40	23.20	8.65					
Mental wandering not related to the topic of the lecture	Pre-test	40	28.27	7.93	39	5.300	Statistically significant (0.01)	0.84	Great impact
	Post-test	40	20.65	7.81					
The scale as a whole	Pre-test	40	55.85	11.93	39	3.957	Statistically significant (0.01)	0.80	Great impact
	Post-test	40	46.87	11.12					

Table 12 demonstrates that the arithmetic mean of the post-test score of the experimental group on the overall mind wandering scale was 46.88 with a standard deviation of 11.12 out of a total possible score of 78. This is lower than their arithmetic mean of the pre-test score, which was 55.85 with a standard deviation of 11.12-a difference of 8.99 points. This indicates that there are differences between the arithmetic mean of the pre-test and post-test scores of the experimental group on the mind wandering scale, in favor of the pre-test. This means that mind wandering decreased after the implementation of the guidance program.

To verify the statistical significance of these differences, the t-value for the paired groups was calculated, as shown in the previous Table 12. The calculated *t*-value was higher than the critical t-value, which is 2.797 at a significance level of (0.01) with 39 degrees of freedom. This indicates statistically significant differences at the (0.01) level across all dimensions of the mental wandering scale, as well as the overall score. Therefore, the hypothesis stating that “there are statistically significant differences between the mean scores of the experimental group in the pre-test and post-test of the mental wandering scale after using the proposed guidance program, favoring the pre-test,” is accepted. This suggests a reduction in mental wandering in the post-test for the experimental group.

To assess the effect size, it was calculated using Cohen’s d for paired groups, as shown in Table 12. The values ranged from 0.60 to 0.84, with the overall effect size for the total score on the mental wandering scale reaching 0.80. This indicates a high effect of the independent variable on the dependent variable, suggesting that the proposed counseling program had a significant and meaningful impact in reducing mental wandering among students in scientific departments at the College of Science and Humanities in Afif. This effect is not only statistically significant but also carries important educational and scientific implications.

### 3.3 Interpretation of the first and second hypotheses results

The results indicate that the proposed counseling program effectively reduced mental wandering in the experimental group compared to the control group, which did not receive any intervention and continued to show high levels of mental wandering. These findings align with those of Al-Feil (2018), who found that scenario-based learning helps reduce mental wandering, and with the study by Qasim et al. (2016), which confirmed the role of deep learning in lowering mental wandering levels.

These results can be attributed to several key factors:

1. The structure and sequence of the counseling program, along with its clearly defined objectives and organized delivery of content, gave students an opportunity to enrich both their direct and indirect experiences. This helped them develop better attention strategies and improve their focus during lectures.
2. The use of both individual and collaborative learning approaches played a significant role in reducing mental wandering, as it catered to diverse learning preferences and encouraged active engagement.
3. Assigning specific tasks to students as part of the counseling program helped direct their thoughts and attention toward performance-based activities, which in turn minimized distractions and off-topic thinking during lectures.
4. The variety of learning resources and technological tools integrated into the program helped capture students’ attention and enhance their mental alertness, ultimately contributing to a decrease in mental wandering.

### 3.4 The third hypothesis

This hypothesis states that:

“There are differences between the mean scores of the experimental and control groups in the post-measurement of the digital stress scale, as a result of using the proposed counseling program”.

To test this hypothesis, the data was described and summarized by calculating the means and standard deviations, followed by conducting an Independent Samples *t*-Test. The results are shown in Table 13.

**Table 13:** Descriptive statistics, *t*-value in post-test between the experimental and control groups, and effect size on the digital stress scale.

Dimensions of the digital stress scale	Group	Number	Arithmetic mean	Standard deviation	Degree of freedom	*t*- test value	Statistical significance	Eta squared ( $\eta^{2}$ )	Effectiveness and impact
Digital social acceptance anxiety	Control	40	28.70	11.01	78	3.828	Statistically significant (0.01)	0.398	Great impact
	Experimental	40	21.02	6.28					
Cyber risks	Control	40	21.42	7.26032	78	3.721	Statistically significant (0.01)	0.294	Medium impact
	Experimental	40	17.35	6.08					
Availability stress	Control	40	16.10	6.27	78	2.952	Statistically significant (0.01)	0.317	Great impact
	Experimental	40	12.55	4.290					
Multitasking	Control	40	9.65	3.88	78	2.935	Statistically significant (0.01)	0.315	Great impact
	Experimental	40	7.65	1.86					
Fear of losing information and communication	Control	40	13.90	5.45	78	4.127	Statistically significant (0.01)	0.423	Great impact
	Experimental	40	9.90	2.790					
The scale as a whole	Control	40	89.77	29.20	78	4.232	Statistically significant (0.01)	0.432	Great impact
	Experimental	40	68.47	12.64					

As shown in Table 13, the mean post-test score of the experimental group on the Digital Stress Scale was 68.475 with a standard deviation of 112.64, out of a maximum possible score of 135. This score is noticeably lower than that of the control group, which had a mean of 89.775 and a standard deviation of 129.20—a difference of 21.3 points. This suggests that the goal was achieved: the experimental group showed a reduction in digital stress levels compared to the control group. This indicates a significant difference between the mean scores of the two groups in the post-test of the Digital Stress Scale.

To verify the statistical significance of these differences, an independent samples *t*-test was conducted for the two groups, which were equal in size, as shown in Table 13. The calculated t-values for all dimensions of the scale, as well as for the overall score, were found to be greater than the critical t-value of 3.106 at 78 degrees of freedom and a significance level of 0.01. This confirms the statistical significance of the differences at the 0.01 level across all dimensions and the total score of the Digital Stress Scale.

Thus, the hypothesis stating that “there are statistically significant differences between the mean post-test scores of the control and experimental groups on the Digital Stress Scale following the implementation of the counseling program” is valid.

To assess the effect size, Eta squared (*η*^2^) was calculated to determine the impact of the independent variable on the dependent variable. The value for the overall scale was 0.43, indicating that 43% of the variance in digital stress scores between the two groups can be attributed to the experimental intervention. Since *η*^2^ exceeded 0.14 across all dimensions, this reflects a large and meaningful effect, with important educational and scientific implications for the effectiveness of the proposed counseling program in reducing digital stress.

### 3.5 The fourth hypothesis

The fourth hypothesis states that:

“There are differences between the mean pre-test and post-test scores of the experimental group on the Digital Stress Scale following the implementation of the proposed counseling program”.

To verify this hypothesis, the data were summarized by calculating the mean and standard deviation, followed by conducting a Paired-Samples *t*-Test to assess the differences between pre- and post-test scores. The results are presented in Table 14.

**Table 14:** Descriptive statistics and *t*-test results for the pre- and post-test scores of the experimental group on the digital stress scale, including effect size calculation.

Dimensions of the digital stress scale	Group	Number	Arithmetic mean	Standard deviation	Degree of freedom	*t*- test value	Statistical significance	D effect value	Effectiveness and impact
Digital Social Acceptance Anxiety	Pre-test	40	27.00	8.62	39	3.775	statistically significant (0.01)	0.6	Medium impact
	Post-test	40	21.02	6.28					
Cyber Risks	Pre-test	40	19.27	6.79	39	2.872	statistically significant (0.01)	0.5	Medium impact
	Post-test	40	17.35	6.08					
availability Stress	Pre-test	40	15.25	5.30	39	3.582	statistically significant (0.01)	0.6	Medium impact
	Post-test	40	12.55	4.29					
Multitasking	Pre-test	40	9.60	3.03	39	4.306	statistically significant (0.01)	0.7	Medium impact
	Post-test	40	7.65	1.86					
Fear of losing information and communication	Pre-test	40	11.85	4.15	39	3.296	statistically significant (0.01)	0.6	Medium impact
	Post-test	40	9.90	2.79					
The scale as a whole	Pre-test	40	82.97	23.95	39	5.239	statistically significant (0.01)	0.83	Great impact
	Post-test	40	68.47	12.64					

As shown in Table 14, the mean post-test score of the experimental group on the Digital Stress Scale was (68.4), with a standard deviation of (12.64), out of a total possible score of 135. This is noticeably lower than their mean pre-test score of (82.97), reflecting a (difference of 14.5 points). This indicates a significant difference between the pre-and post-test scores of the experimental group in favor of the pre-test, suggesting a reduction in digital stress levels after the application of the program.

This decline can be attributed to the experimental treatment, in this case, the implementation of the counseling program.

To assess the significance of these differences, a Paired-Samples *t*-Test was conducted, as presented in Table 14. The calculated *t*-values exceeded the critical *t*-value of 2.797 at 39 degrees of freedom and a significance level of 0.01. This confirms the presence of statistically significant differences at the 0.01 level in both the overall scale and all its sub-dimensions. Therefore, the hypothesis stating that *“there are statistically significant differences between the pre- and post-test mean scores of the experimental group on the Digital Stress Scale after applying the proposed counseling program, in favor of the pre-test”* is accepted. This supports the conclusion that digital stress was reduced in the post-test as a result of the intervention.

To measure the impact of the intervention, Cohen’s *d* was used to calculate effect size for the paired groups. While the effect size for individual dimensions of the scale was moderate, the value for the overall scale reached 0.83, indicating a large effect. This suggests that the independent variable (the counseling program) had a strong influence on the dependent variable (digital stress), highlighting the significant educational value and scientific relevance of applying the proposed counseling program to reduce digital stress across all its dimensions among students in the scientific departments at the College of Science and Humanities in Afif.

### 3.6 Interpretation of the results of the third and fourth hypotheses

The findings related to the third and fourth hypotheses indicate that the proposed counseling program had a significant impact in reducing digital stress among students in the experimental group compared to the control group, which did not receive any experimental treatment.

The researcher attributes these results to several key factors:

1. *Clear program objectives*: The counseling program had well-defined goals that helped students in the experimental group understand what digital stress is, along with its causes, symptoms, and psychological impacts on student life. This awareness enabled them to address the sources of their digital stress and adopt protective strategies to manage it.
2. *Content of the counseling sessions*: The sessions emphasized the negative psychological, social, and emotional effects experienced by students with high levels of digital stress. The program provided appropriate scientific content to address these issues and gave students the opportunity to explore and listen to real-life experiences of individuals who had suffered from digital stress, which had a meaningful impact on their lives.
3. *Activities and exercises*: The program included activities that required students to engage in positive social interaction through group work, encouraging them to abandon harmful digital habits. These practices helped students feel more relaxed and reduce anxiety and fear related to the psychological aspects of digital stress.

### 3.7 The fifth hypothesis

The fifth hypothesis states:

“There is a relationship between mind wandering and digital stress among students in the experimental group after applying the counseling program”.

To test this hypothesis, Pearson’s correlation coefficient (r) was calculated to determine the type and strength of the relationship between the two variables. The results are presented in Table 15.

**Table 15:** The Pearson correlation coefficient r mental wandering and digital stress.

Variable	Correlation r	Digital stress
Mental wandering	Pearson’s correlation coefficient (r)	+0.311
	Significance level	+0.05
	Experimental sample size	40

Table 15 demonstrates that the Pearson correlation coefficient equals 0.311 with a significance level of 0.05, indicating a weak positive correlation between digital stress and mind wandering among the research sample of students from the scientific departments at the College of Science and Humanities in Afif.

This result can be interpreted as the proposed counseling program helping students reduce both mind wandering and digital stress. As digital stress decreases, mind wandering also tends to decrease, and vice versa. The program provided guidance and instructions to reduce stress, which in turn helped students reduce mind wandering during lectures, improving their focus and learning outcomes.

## 4 Conclusion

This study investigated the effectiveness of a proposed counseling program in reducing mental wandering and digital stress among students in the Scientific Departments at Shaqra University in Afif, Saudi Arabia. Utilizing a descriptive and quasi-experimental design, the research was conducted on a purposive sample of 80 students who exhibited high levels of mental wandering and digital stress. The sample was divided equally into a control group and an experimental group, with the latter participating in a seven-session counseling program.

The counseling program proposed by the study is different from those in previous studies in that it caters for the needs of Saudi students and considers the cultural and social sensitivities of the Saudi society. It was designed specifically for students who live in a Saudi context. It also avoided the deficiencies demonstrated by previous programs.

The results of the research showed that the proposed counseling program was effective in reducing both mental wandering and digital stress in students. The data suggests that there is a positive correlation between mental wandering and digital stress, which means that a decrease in the level of digital stress is accompanied by a decrease in mental wandering, and vice versa. This is because the program provided practical guidance and guidance to reduce digital stress, which in turn contributed to reducing mental wandering within lectures. This reduction in mental wandering allows students to better focus on the course content and improve their academic achievement. Therefore, the proposed counseling program is an effective pedagogical tool to support students in managing concentration, reducing mental stress, and enhancing the quality of learning within the lecture environment.

Considering these findings, the researcher recommends several key steps to enhance student well-being and support improved learning outcomes. First, to reduce mental wandering during lectures, instructors should consider breaking content into smaller, more manageable segments and incorporating a variety of engaging methods, such as enrichment activities and group work, to maintain student focus. Second, promoting a healthy and balanced use of technology is essential; educators should guide students in using digital tools in ways that support learning without compromising their physical, mental, or social health. Third, student’s at all educational levels should have access to psychological and social support, including school counseling services and community programs. These resources can help students use their free time more productively, counteract the negative effects of excessive internet use, and raise awareness about digital addiction. Finally, offering training sessions and workshops for teachers, administrators, and support staff can equip them with the necessary skills to address the educational and psychological challenges students face in today’s digital environment.

Building on the results of this study, there are several meaningful directions future research could take. One area worth exploring is how digital stress affects students’ academic performance at different educational levels, which could help us better understand its real impact on learning and success. Another valuable focus would be looking into how different teaching methods might help keep students more engaged and reduce mental drifting during lessons. It would also be helpful to examine whether levels of digital stress vary depending on factors like gender, field of study, or academic performance, so we can identify which groups might need more support. Lastly, developing a model that looks at the link between academic perseverance and digital stress could offer useful insights into how to build student resilience in today’s tech-heavy learning environments.

## Data Availability

The original contributions presented in the study are included in the article/Supplementary material, further inquiries can be directed to the corresponding author/s.
