# Supplementary material for: The effectiveness of a proposed counseling program in reducing mental wandering and digital stress among the students of the scientific departments in Afif, Shaqra University
Source: Front Psychol. 2026 Apr 22;17:1739002. doi: 10.3389/fpsyg.2026.1739002 (PMC13143759; doi:10.3389/fpsyg.2026.1739002)
Supplement: Supplementary file 1 [file Supplementary_file_1.pdf]

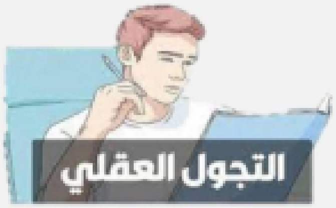

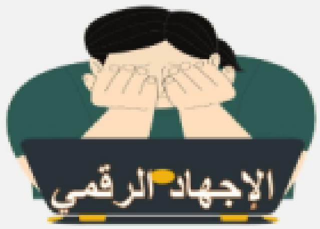

تشخيص التجول العقلي والإجهاد الرقمي لدى طلاب الجامعة.

عزيزي الطالب/ نأمل منك التعاون معنا في تعبية هذا الاستبيان لتشخيص جوانب الإجهاد الرقمي والتجول العقلي والتي تساعدنا على تقديم الإرشادات المناسبة للتخلص من هذه الأعراض لديك.

ويعرف الاجهاد الرقمي: بأنه ما يدركه الفرد من مظاهر الضيق والإرهاق والشعور بالقلق والتعب الذي يشعر به الأفراد نتيجة عدم التكيف مع متطلبات التكنولوجيا الرقمية.

ويعرف التجول العقلي: بأنه تحول تلقائي في الانتباه العقلي من المهمة الأساسية إلى أفكار أخرى مشتتة داخلياً أو خارجياً قد تكون هذه الأفكار مرتبطة بالمهمة نفسها أو غير مرتبطة بها.

dr.khalid122@gmail.com Switch account

Not shared

* Indicates required question

الاسم اختيارياً

Your answer

الكلية: *

Choose

This is a required question

القسم العلمي *

Choose

This is a required question

المستوى الدراسي: *

Choose

المعدل التراكمي: *

Choose

Next

Clear form

مقياس الإجهاد الرقمي: (إعداد الباحث)

البعد الأول: قلق القبول الاجتماعي الرقمي *

	موافق بشدة	موافق	أحياناً	غير موافق	غير موافق بشدة
أحتاج أن أبدو مثاليًا في مشاركاتي الرقمية.	○	○	○	○	○
أتردد في نشر مشاركات رقمية بسبب الخوف من ردود الفعل السلبية.صف 2	○	○	○	○	○
أشعر بالخجل بشأن ردود الفعل التي قد أتلقاها على مشاركاتي الرقمية.	○	○	○	○	○
أشعر بالقلق بشأن ما يعتقده الآخرون عني بناءً على	○	○	○	○	○

أشعر بالقلق من مشاركاتي الرقمية بعد نشرها.					
اتجنب نشر المشاركات الرقمية.					
أحذف المشاركات الرقمية بعد نشرها.					
اشعر بالخوف من التحدث أمام الآخرين عبر الإنترنت.					
أشعر بالانزعاج أو القلق عند استخدام التكنولوجيا الرقمية.					

البعد الثاني: المخاطر السيبرانية: *

	موافق بشدة	موافق	أحياناً	غير موافق	غير موافق بشدة
أشعر بالقلق بشأن سرقة معلوماتي الشخصية وهويتى الرقمية عبر الإنترنت.					
أشعر بالقلق بشأن تعرضي للمضايقات أو الإبتزاز الإلكتروني الإساءة عبر الإنترنت.					
أشعر بالقلق بشأن تعرض أجهزتي أو حساباتي للهجوم عبر الإنترنت.					
أشعر بالقلق بشأن تعرضي لفيروسات أو برامج ضارة عبر الإنترنت.					

أشعر بالقلق بشأن فقدان الوصول إلى معلوماتي أو بياناتي عبر الإنترنت.	○	○	○	○	○
أشعر بالقلق بشأن فقدان السيطرة على حياتي بسبب المخاطر السيبرانية.	○	○	○	○	○

البعد الثالث: إجهاد التوافر والإتاحية: *

	موافق بشدة	موافق	أحياناً	غير موافق	غير موافق بشدة
أشعر بالضغط أن أكون متاحًا دائمًا للرد على الرسائل أو المكالمات الرقمية.	○	○	○	○	○
أشعر بالذنب إذا لم أرد على الرسائل أو المكالمات	○	○	○	○	○
أشعر بالذنب إذا لم أرد على الرسائل أو المكالمات الرقمية على الفور.	○	○	○	○	○
أشعر بالقلق إذا تأخرت في الرد على الرسائل أو المكالمات الرقمية.	○	○	○	○	○
أشعر بالإرهاق بسبب كثرة الرسائل أو المكالمات الرقمية التي أتلقاها.	○	○	○	○	○
أجد صعوبة في التركيز بسبب كثرة الرسائل أو المكالمات الرقمية التي أتلقاها.	○	○	○	○	○

* :البعد الرابع: تعدد المهام

	موافق بشدة	موافق	أحياناً	غير موافق	غير موافق بشدة
أجد صعوبة في التركيز على أكثر من مهمة واحدة في نفس الوقت باستخدام التكنولوجيا الرقمية.	○	○	○	○	○
أشعر بالارتباك بسبب كثرة الرسائل أو النوافذ التي تظهر على جهازي الرقمي.	○	○	○	○	○
أشعر بالإرهاق بسبب محاولة القيام بمهام متعددة في نفس الوقت باستخدام التكنولوجيا الرقمية.	○	○	○	○	○

* البعد الخامس: الخوف من فقد المعلومات والاتصال

	موافق بشدة	موافق	أحياناً	غير موافق	غير موافق بشدة
أشعر بالقلق بشأن فقدان الاتصال بالأخرين بسبب استخدام التكنولوجيا الرقمية.	○	○	○	○	○
أشعر بالقلق بشأن فقدان المعلومات المهمة بسبب استخدام التكنولوجيا الرقمية.	○	○	○	○	○
أشعر بالقلق بشأن فقدان السيطرة على حياتي بسبب استخدام التكنولوجيا الرقمية.	○	○	○	○	○

أشعر بالخوف من فقد العلاقات التي لدي مع الآخرين عبر الإنترنت.	○	○	○	○	○
